# Case of Rupture of the Linea Alba in Pregnancy

**Published:** 1826-07

**Authors:** 


### RUPTURE OF THE LINEA ALBA.

In the May Number of the Repository a case of this kind, occurring during pregnancy, is related by Mr. Dendy.

Mr. D. makes some prefatory remarks on muscular contractility, ruptures of tendons, &c. which, though they may be very true, are certainly any thing but very new. But to the case.

Mrs. Parsons, ætat. 28, three days after a very severe labour, in which the vectis was employed, felt acute pain about the umbilicus and pubes with smarting in the vagina on the evacuation of urine; both this and the lochia were abundant. On examination there was found a large irregular tumour, between the umbilicus and cephoid cartilage, evidently a protrusion of the intestines. After taking food, this protrusion was increased, and the peristaltic motion very discernible in it. On reducing the hernia a considerable rent in the linea alba was felt. Ten leeches and a blister were applied near the part: and the pain was relieved. Next morning inflammatory symptoms re-appeared, the applications were repeated with frequent doses of hydr. subm. and pulv. antim. with good effect. It was now found that she had a slight prolapsus of the uterus. A broad flannel bandage was applied. In the evening there were nearly two ounces of pus discharged from the vagina. At the expiration of a month, the protrusion was gone—bandage still worn. The prolapsus uteri troublesome at times.

Mr. Dendy suggests the general use of a bandage around the abdomen during labour. It would probably do no harm, and it is a query whether it would do much good.

